# The Emerging Role of MMP12 in the Oral Environment

**DOI:** 10.3390/ijms24054648

**Published:** 2023-02-28

**Authors:** Bingpeng Lin, Hooi Leng Ser, Lijing Wang, Jiang Li, Kok-Gan Chan, Learn-Han Lee, Loh Teng-Hern Tan

**Affiliations:** 1 Novel Bacteria and Drug Discovery Research Group (NBDD), Microbiome and Bioresource Research Strength (MBRS), Jeffrey Cheah School of Medicine and Health Sciences, Monash University Malaysia, Bandar Sunway 47500, Selangor Darul Ehsan, Malaysia; 2 Department of Orthodontics, Affiliated Stomatology Hospital of Guangzhou Medical University, Guangzhou 510180, China; 3 Guangzhou Key Laboratory of Basic and Applied Research of Oral Regenerative Medicine, Guangzhou 510182, China; 4 Department of Biological Sciences, School of Medical and Life Sciences, Sunway University, Bandar Sunway 47500, Malaysia; 5 Vascular Biology Research Institute, School of Life Sciences and Biopharmaceutics, Guangdong Pharmaceutical University, Guangzhou 510006, China; 6 Division of Genetics and Molecular Biology, Institute of Biological Sciences, Faculty of Science, University of Malaya, Kuala Lumpur 50603, Malaysia; 7 International Genome Centre, Jiangsu University, Zhenjiang 212013, China; 8 Innovative Bioprospection Development Research Group (InBioD), Clinical School Johor Bahru, Jeffrey Cheah School of Medicine and Health Sciences, Monash University Malaysia, Johor Bahru 80100, Malaysia

**Keywords:** MMP12, tissue expression, periodontitis, bone remodelling, OTM, OSCC

## Abstract

Matrix metalloproteinase-12 (MMP12), or macrophage metalloelastase, plays important roles in extracellular matrix (ECM) component degradation. Recent reports show MMP12 has been implicated in the pathogenesis of periodontal diseases. To date, this review represents the latest comprehensive overview of MMP12 in various oral diseases, such as periodontitis, temporomandibular joint dysfunction (TMD), orthodontic tooth movement (OTM), and oral squamous cell carcinoma (OSCC). Furthermore, the current knowledge regarding the distribution of MMP12 in different tissues is also illustrated in this review. Studies have implicated the association of MMP12 expression with the pathogenesis of several representative oral diseases, including periodontitis, TMD, OSCC, OTM, and bone remodelling. Although there may be a potential role of MMP12 in oral diseases, the exact pathophysiological role of MMP12 remains to be elucidated. Understanding the cellular and molecular biology of MMP12 is essential, as MMP12 could be a potential target for developing therapeutic strategies targeting inflammatory and immunologically related oral diseases.

## 1. Introduction

Belonging to a group of over 20 zinc-dependent proteases, matrix metalloproteinases (MMPs) are capable of degrading different types of ECM proteins. MMPs have been known to have a major role in keeping mucosal integrity and regulating diverse pathological processes, including inflammation, cancer, and fibrosis. MMP12 is an extracellular MMP that was first found to be expressed in alveolar macrophages of smokers and then assigned to the matrix metallopeptidase family due to comparable genomic structure and activity [1]. Full-length MMP12 includes three unique domains: an amino-terminal propeptide domain that could regulate enzyme latency, a zinc- and calcium-binding catalytic domain, and a carboxy-terminal haemopexin-like domain that determines substrate selectivity [2]. Like other MMPs, MMP12 is found in a cluster of genes encoding MMP on chromosome 11q22.3 [3]. Released as a 54-kDa pro-form enzyme, MMP12 was autolyzed to produce 45-kDa and 22-kDa products [4]. Aside from auto-proteolytic processing, the MMP12 enzyme may activate additional MMP, which in turn can activate pro-MMP1 and pro-MMP9 [5]. It can also destroy a wide range of ECM components, such as type IV collagen, fibronectin, laminin, vitronectin, chondroitin sulphate, and heparan sulphate proteoglycan [4,6,7]. Interestingly, intracellular MMP12 in macrophages was shown to kill bacteria directly via the carboxy-terminal domain and was characterized as a novel antimicrobial peptide in nature [2].

In both experimental and clinical research, MMP12 has been identified as a crucial component in various normal physiological and pathological conditions. The functions of MMP12 include: (i) regulatory functions in embryonic development, reproduction, and tissue remodelling; (ii) an invasive role by degrading the basement membrane, allowing macrophages to enter wounded tissue during inflammation [8,9]; (iii) a significant damaging effect in pulmonary emphysema [10,11]; (iv) a wound-healing repair role [12,13]; and (v) anti-tumorigenic, anti-inflammatory, antibacterial, and antiviral properties [2,14,15,16]. Given its wide range of functions, MMP12 has been associated with many significant human diseases [17], including inflammatory diseases [11,18,19,20,21], different types of cancers [22,23,24,25] and vascular diseases [26], and brain-related disorders [27,28,29].

So far, research on the role of MMP12 in the oral environment is scarce, despite several studies having been undertaken to evaluate its role in systemic disease. To better elucidate the possible involvement of MMP12 in a variety of disorders, particularly those affecting the oral cavity, this review summarises the MMP12 expression levels in normal oral tissues and the advanced research into tissue remodelling and oral diseases. Furthermore, MMP12 has been explored as the molecular target for the diagnosis and treatment of oral diseases.

## 2. Expression of MMP12 in Different Tissues and Cell Types

MMPs (particularly MMP12) can be regarded as risk factors for oral diseases, and their levels can be valuable symbols for early diagnosis and assessment of prognosis [30]. However, the physiological functions of MMP12 are not well known. To better understand its possible roles associated with various diseases, it is critical to explore the MMP12 expression profiles in different kinds of tissues and cell types.

### 2.1. Expression of MMP12 in Normal Human Tissues

**Figure 1:**
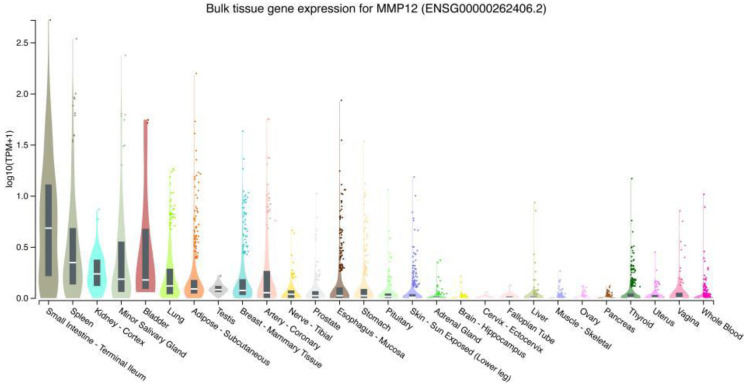
Violin plot of MMP12 expression in normal human tissues, coloured by organs. Bulk tissue gene expression for MMP12 from GTEx portal (https://gtexportal.org/home/gene/MMP12 (accessed on 25 March 2022)).

The MMP12 mRNA and protein expressions are regulated by the stage of cellular development [4]. Given that it is undetectable in the circulating monocytes, MMP12 was formerly thought to be restricted to tissue macrophages [8]. However, it was later discovered to be significantly regulated by several cytokines and growth factors [31]. In general, it is widely accepted that MMP12 is not detectable in adult tissue and has a restricted cell distribution in its normal state. According to these findings, Pagenstecher and Unemori et al. concluded that, under normal circumstances, humans do not secrete MMP12, or only secrete a negligible amount [32,33]. In contrast, the GTEx project’s mRNA sequencing dataset suggested that MMP12 exists in normal tissues, such as the small intestine, spleen, minor salivary gland, bladder, and lung, but not in the uterus, vagina, or blood. Although the mean level of expression is not particularly high (as illustrated in Figure 1), a plausible explanation for these discrepancies could be due to the different sensitivities of the various experimental procedures. Additionally, MMP12 expression has been documented in tissues that undergo fast remodelling, such as the term placenta during human foetal development, and in several cancer tissues [34,35]. Increasing MMP12 expression was also demonstrated in a variety of tumour and inflammatory tissues compared to normal tissues [36,37,38,39]. Despite its prevalence in inflammatory and cancer tissues, these results surmise that MMP12 was only detected in a few types of normal tissues, as well as the fast-remodelling tissue, but their expressions were relatively low.

In terms of the expression of MMP12 in different cell types, this enzyme was thought to be expressed in a variety of cell types, including monocytes, osteoclasts, endothelial cells, corneal epithelial cells, smooth muscle cells, and chondrocytes [40]. However, according to the study of Hou et al., MMP12 existed in a limited number of cells, such as tissue macrophages and hypertrophic chondrocytes [41]. Both MMP12 mRNA and protein were detected in stromal cells of placental tissue and macrophages, using in situ hybridization and immunohistochemistry [1]. Apart from macrophages, altered epithelial cells in skin cancer have been shown to express MMP12 [23]. Its expression level was demonstrated to be associated with epithelial dedifferentiation and histological aggressiveness, implying that MMP12 produced by epithelial cells functions differently than macrophage-secreted MMP12 [35]. To further investigate the cell distribution of MMP12, we conducted a systematic search using the Human Protein Atlas (HPA) single-cell RNA sequencing (scRNA) database and discovered that it was enriched in extravillous trophoblasts expressed in blood macrophage, endocrine glandular epithelial cells, and placental Hofbauer cells. MMP12 was shown to be poorly represented in urothelial cells, B cells, ductal cells, and basal prostatic cells in HPA datasets. Due to its capacity to profile gene expression for all cell types in many tissues unbiasedly and at a high resolution, the scRNA sequencing method has been widely employed in various studies. Thus, our findings implied that MMP12 is primarily expressed in the small intestine, spleen, minor salivary gland, bladder, and lung in normal tissue, whereas macrophages, trophoblasts, and endothelial cells are predominantly expressed in abnormal tissue. Additionally, it is worth noting the diverse MMP12 expressions within the same species for a given cell type. Furthermore, when cells and tissues are inflamed or proliferate abnormally, MMP12 expression is greatly elevated.

### 2.2. MMP12 in Oral Tissues

**Figure 2:**
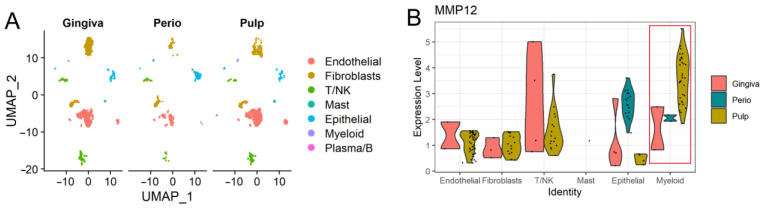
scRNA-Seq analysis of oral tissues from GEO datasets (GSE161266, GSE164241). The data used for this research were downloaded from online public database, and Seurat R package (version 4.0.2) was used for downstream analysis. (**A**) UMAP plot of the gingiva, periodontal ligament (Perio), and pulp tissues. (**B**) Violin plot of MMP12 expression in different cells of dental tissues, coloured by tissues.

Although MMP12 is expressed in a variety of systemic organs, its expression profile in oral tissues is still unknown. Previous work has shown that MMP12 can be found in healthy persons’ saliva and gingival crevicular fluid (GCF), but this does not imply tissue expression in the oral cavity. Several studies indicated that MMP12 mRNA was expressed in salivary glands, tongue, gingival tissues, and periodontal ligament (PDL), but not in pulp tissues [42]. In addition, the status of MMP12 expression at the single-cell level in oral tissues is yet to be elucidated. To ascertain the expression of MMP12 in various healthy oral tissues and cell types, single-cell RNA sequencing datasets from the Gene Expression Omnibus (GEO) database were retrieved for analysis using the R programming language. As illustrated in Figure 2, MMP12 is expressed in healthy gingiva, periodontal ligament, and even pulp tissues, and especially highly expressed in dental pulp myeloid cells and epithelial cells, which contradicts the previous results [42]. Relatively low MMP12 expression is seen in the fibroblast, B cells, and mast cells of dental tissues. These results indicated the potential role of MMP12 in oral tissue homeostasis and the regulation of immune function.

## 3. Role of MMP12 in the Oral Environment, Diseases, and Cancers

MMP12 has been demonstrated to be an inducer and regulator of the inflammatory process associated with oral mucosal disorders. Moreover, it is involved in the development, remodelling, and degeneration of oral tissues. However, understanding MMP12’s role in oral tissue remodelling and the pathogenesis of numerous oral ailments is still elusive. Pharmacological therapy options can only be improved if the underlying mechanism of these diseases is elucidated. Therefore, the following section investigates the expressions and role of MMP12 in periodontal tissue remodelling and dental disorders. Table 1 summarizes studies investigating the involvement of MMP12 in oral diseases.

### 3.1. MMP12 in Periodontal Disease

Dental inflammatory diseases linked with irreversible tissue loss are frequently caused by dysregulated host inflammatory responses and dysbiotic microbiome, as well as the excessive synthesis of tissue-remodelling enzymes, particularly MMPs [59,60]. MMP12 cleaves elastin and other extracellular matrix components required for oral epithelial integrity [36]. However, the relationship between MMP12 and the tissue degradation associated with chronic periodontitis remains uncertain. Recent studies have established a link between MMP12 and periodontal tissue degradation. MMP12 levels in saliva may reflect several features of periodontal inflammation. MMP12 levels were significantly greater in those with more severe periodontal inflammation [52]. Furthermore, the MMP12 expression level in the gingival crevicular fluid (GCF) of patients with aggressive periodontitis was reduced after periodontal treatment [54]. This indicates that MMP12 may be a risk factor for periodontal disease. Moreover, a recent study discovered that MMP12 was highly overexpressed in chronic periodontitis tissue [36]. The existence of MMP12-producing monocytes and decreased tropoelastin levels in periodontitis patients’ inflamed gingival tissue was determined as a novel route contributing to periodontal aetiology. Thus, the increased expression of MMP12 in the GCF, saliva, and gingival tissues of periodontal disease patients suggests that this enzyme may be helpful as a diagnostic marker of disease status and a predictor of future disease.

However, MMP12 expression, on the other hand, was found to follow a distinct pattern in other periodontal diseases. Kang et al. found that the level of MMP12 expression was revealed to be 96 times lower in idiopathic gingival fibromatosis (IGF) tissues than in normal gingival tissues [57]. The study demonstrated that low MMP12 expression impairs protein hydrolysis in IGF gingival tissue, contributing to the enlargement of the gingiva. Interestingly, MMP12 and MMP1 were significantly upregulated in periodontitis patients’ gingival overgrowth tissues compared to healthy subjects’ control gingival tissues, owing to these enzymes’ analogous functions, implying that they both participate in the pathological metabolism of periodontal connective and skeletal tissues [53]. The differential expression of MMP12 in hyperplastic gingival tissue may reflect the periodontal tissues’ inflammatory condition.

Kim et al. studied the genetic alterations in aged periodontal tissue at the transcriptome level and discovered that MMP12 expression was increased in aged gingival tissues, implying that increased MMP12 expression is a molecular signature of natural gingival ageing [58]. In contrast to this investigation, Holmström et al. discovered that salivary MMP12 levels were considerably lower in persons aged 40–64 compared to those aged less than 40 years [52]. This could be explained by discrepancies between expressions of mRNA and protein since mRNA accounts for only a portion of protein differences, which is further influenced by translation and degradation processes. In this regard, MMP12 may play a role in the periodontium age-related cellular and clinical alternations [58]. It must be borne in mind that the expression of MMP12 in saliva and tissues does not always imply enhanced enzyme activity in the tissue. As a result, a functional clinical study strategy is preferable for elucidating MMP12’s precise role in periodontal diseases.

**Figure 3:**
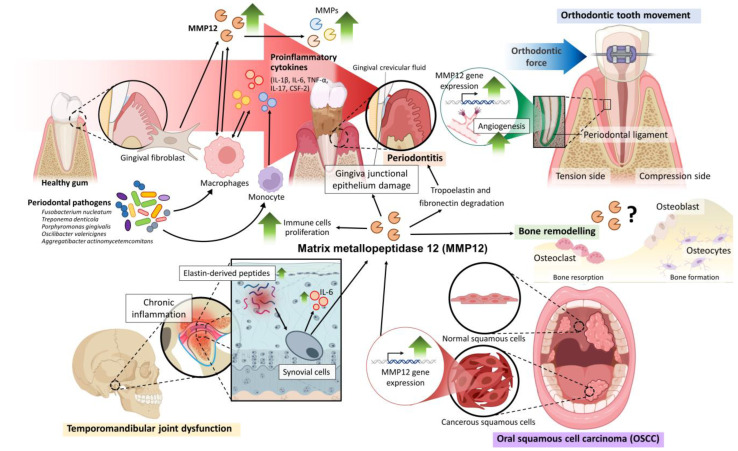
The role of MMP12 in the oral environment and the proposed mechanisms of MMP12 in the pathologies of oral disorders, including periodontitis, temporomandibular joint dysfunction, and oral squamous cell carcinoma. The potential involvement of MMP12 in orthodontic tooth movement and bone remodelling is also included. This figure was created with the help of BioRender.com.

Although the specific mechanisms underlying the pathological levels of MMP12 synthesis in periodontal tissue are unknown, various inflammatory pathways may be involved (Figure 3). Among the different cytokines detected in dental pulp during inflammation, TNF-α and IL-1β enhanced the expression of MMP12 in dental pulp and gingival fibroblasts [61,62]. Primarily, these cytokines were produced in response to the lipopolysaccharides of bacteria associated with periodontal disease. Furthermore, the synthesis of MMP12 was induced by colony-stimulating factor 2 (CSF-2) stimulated in monocyte-derived cells [36], which is in contrast to other MMPs that are activated via the COX2/PGE2 pathway [63]. In a more recent study, similar group of researchers found that CSF-1 receptor also regulates expression of MMP12. In periodontitis patients, MMP12 production and other inflammatory mediators were attenuated in response to the blocking of CSF-1 receptor (CSF-1R) in the inflamed gingiva and circulating monocytes [39]. Nevertheless, more in-depth study is needed to clarify the intricate mechanisms of the pro-inflammatory stimuli in regulating the expression of MMP12 from gingival tissue and immune cells.

Similar to the functions of other MMPs in the pathogenesis of chronic periodontitis, the production of MMP12 can cause damage to periodontal tissue. Notably, studies conducted through the MMP12 deletion mice model revealed decreased expression of MMP13 and MMP9 [64], indicating that MMP12 may have an important role in regulating the production of other MMPs. Additionally, MMP12 was identified as a critical regulator in the degradation of intestinal basement membrane laminin and macrophage transmigration, which resulted in the loss of the intestinal tight junction barrier associated with intestinal inflammation [9]. Due to the comparable structure of gingiva junctional epithelium and intestinal epithelium, MMP12 is hypothesized to act as a regulator of macrophage transmigration, resulting in gingiva junctional epithelium damage. In the aspect of inflammatory functions of MMP12 in macrophages, MMP12 was demonstrated to regulate multiple pro-inflammatory effector functions of macrophages in an LPS-induced inflammation mice model. The findings of the study suggested that MMP12 can modulate the proliferation of macrophages and the production of inflammatory cytokines, such as IL-1β, IL-6, TNF-α, CXCL1, and CXCL3, via the ERK/P38 MAPK signalling pathway [65]. A recent bioinformatic study identified that MMP12 is one of the hub genes that mediate the inflammatory process in periodontitis through the IL-17 signalling pathway [51].

In the last decade, increasing evidence shows that the dysbiosis of the oral microbiome is associated with the pathogenesis of periodontal diseases, whereby a complex interplay between dysbiotic microbial communities and aberrant immune responses exists within gingival and periodontal tissues [66,67]. There were studies reporting the effects of oral microbiota related to periodontal diseases in triggering inflammatory responses that increase levels of pro-inflammatory cytokines and MMPs [68]. For example, *Fusobacterium nucleatum*, which was shown to play a significant role in periodontal changes from gingivitis to severe forms of periodontitis, can upregulate IL-8, MMP9, MMP13, and other MMPs [69,70]. Another study also showed that *Treponema denticola*, another periodontal pathogen, together with its effector protein, dentilisin, mediated the upregulation of MMP levels in periodontal tissues among periodontitis patients [71]. As for MMP12, a recent study identified that the TLR2 ligands from a specific gut microbe, *Oscilibacter valericignes*, increased the production of MMP12 by adipose tissue macrophages via a MYD88-ATF3-dependent signalling pathway [72]. However, only a limited study reported the direct impact of oral microbiota on MMP12 expression in the oral environment. *Porphyromonas gingivalis* is one of the major periodontal pathogens that was found to increase the expression of MMP12 in gingival fibroblasts [55]. Moreover, the production of MMP12 could be driven by the secretion of pro-inflammatory cytokines within the oral tissue via a microbiota-dependent mechanism. For instance, *Aggregatibacter actinomycetemcomitans* promoted the expression of CSF-2 in the gingival epithelium cells [73], which may drive the production of MMP12 in periodontitis [36].

In an earlier study, MMP12 was reported to function as an antimicrobial peptide against invading pathogens, where intracellular stores of MMP12 are mobilized to phagolysosomes in macrophage and adhere to bacterial cell walls, causing bacterial death [2]. Nonetheless, it remains unknown whether MMP12 can directly destroy periodontal pathogens during the early stages of periodontal diseases, as dysbiosis is closely associated with the development of periodontitis. Nevertheless, further research is needed to disclose the underlying molecular mechanism of MMP12 in periodontal disease.

### 3.2. MMP12 in Bone Remodelling

MMP12 has been linked to pathological connective and osseous tissue metabolisms in periodontal disease patients [53]. Despite the fact that periodontal disease is characterized by bone degradation, the effect of MMP12 on alveolar bone loss is poorly understood. Only a few cells involved in bone remodelling, such as macrophages, chondrocytes, and osteoclasts, were found to generate MMP12 in mouse bone tissue [41]. MMP12 was found to degrade bone at the same rate as MMP9 and MMP14 in specialized bone resorption mouse models, indicating that it plays a minor role in osteoclast migration or bone resorption, despite the fact that it can cleave bone matrix proteins required for matrix and osteoclast interactions [74]. Consistent with this study, MMP12 is released by osteoclasts, but does not efficiently hydrolyse native type I collagen, a significant component of dentin and bone matrixes [75,76,77,78]. It is possible that the critical MMP has not been found yet or is osteoclastic. Furthermore, MMP12 was discovered in human rheumatoid arthritis (RA) osteoclasts. MMP12 expression is increased in rheumatoid arthritis synovial tissues and synovial fluids, implying that this protein is associated with the aetiology of RA [19]. Although this enzyme may not be directly implicated in the resorption of bone, numerous data indicate that it may contribute to the damage to cartilage [78,79]. MMP12-producing osteoclasts may play a role in inflammation, cartilage degradation, and bone destruction in RA. Significantly increased mRNA MMP12 levels were found in the periprosthetic tissues of an abnormal artificial joint, suggesting that pathologic ECM breakdown and tissue remodelling around prostheses may contribute to osteolysis.

In addition, the results revealed that this enzyme might be involved in the deterioration of the bone matrix around the loose hip prosthesis, but its precise role remains unknown [80]. MMP12 may or may not have a role in normal or inflammatory alveolar bone remodelling. Further information about the exact role of MMP12 in bone resorption still is required. MMP12, on the other hand, increased the production of pro-osteogenic factors, such as RUNX-2, BMP-2, and ALP, as well as the creation of calcium deposits [81]. MMP12 is thought to have a critical role in extracellular matrix remodelling during foetal bone development, based on mRNA and immunostaining results from in vitro experiments [35]. An interesting survey conducted by Marc Fajardo et al. revealed that the mRNA expression of MMP12 was dramatically increased in human fracture nonunion tissue when compared to local mineralized callus from the same site, and this enzyme was confirmed to directly bind to, degrade, and inactivate bone morphogenetic protein-2. These findings may also suggest that MMP12 is involved in the nonunion of atrophic bone fractures [82]. Other studies concluded that MMP12 was a new protein associated with bone remodelling, confirming prior findings [83]. Nonetheless, Xin et al. used Mendelian randomization to study the causative relationships between bone mineral density (BMD) and MMPs, specifically MMP1. The findings demonstrated that there is no evidence in the European population for causal effects of MMP expression on BMD measurement in three common areas. Although this study examined the influence of circulating MMP levels on bone mineral density, the intracellular function of MMPs cannot be denied [84]. To our knowledge, it is uncertain whether MMP12 has a role in normal or inflammatory alveolar bone remodelling. MMP12 is presumed to be important in the development of periodontal tissues, and its role in this process should be investigated further. Thus, elucidating the precise role of MMP12 in periodontal tissue growth and bone remodelling requires further investigation.

### 3.3. MMP12 in Orthodontic Tooth Movement

Orthodontic tooth movement results in a significant modification of the ECM in the periodontal ligament as a result of applied orthodontic stresses. The orthodontic tensile strain causes angiogenesis via Collagen IV breakdown in the basement membrane of vascular endothelial [40]. Consistent with this work, the only statistically significant variation in MMP12 levels 24 h after activation of periodontitis teeth undergoing orthodontic therapy was observed [45]. In addition, MMP12 mRNA expression was shown to be much higher in the periodontal ligaments of premolars with occlusal contact compared to third molars executing vertical movement, offering new insight into the molecular process behind vertical tooth movement [48]. Mechanical loading, on the other hand, increases MMP12 expression in chondrosarcoma cells, the rat intervertebral disc, and tail growth plates [85,86]. In contrast, another study found that the mean level of MMP12 in GCF during OTM did not differ substantially between the test and control samples [47,48]. MMP12 expression was increased in response to sulforaphane-induced reductions in orthodontic rotation relapse [46]. We can speculate that MMP12 may play a critical function in the movement of force-applied teeth, but the potential mechanism is still not thoroughly studied. The level of MMP12 production variances may be determined by the manner of tooth movement and individual situations.

### 3.4. MMP12, the Connection with Oral Squamous Cell Carcinoma (OSCC)

MMP12 is involved in the genesis and progression of tumours, specifically in the proliferation, migration, invasion, and metastasis of cancer cells [87,88,89]. Moreover, it has been found to be overexpressed in a variety of human gastrointestinal and lung malignancies [38,90,91,92]. When focusing on the role of MMP12 in oral cancer, Zohra et al. discovered that considerably increased MMP12 expression occurs as a healthy patient progresses to oral submucous fibrosis (OSF) and OSCC. This investigation also found that OSCC patients expressed more MMP12 than OSF patients [49]. In line with this research, another study revealed that the MMP12 levels in the saliva of the OSCC patients were considerably higher than in the healthy and oral benign mass groups. In addition, MMP12 expression was reduced in metastatic OSCC cancers compared to nonmetastatic OSCC tumours [93]. The absence of MMP12 from oral epithelium may be considered a beneficial prognostic protein of oral carcinoma with no invasion [94]. Hence, we may deduce that determining salivary MMP12 levels may be a good, non-invasive, early diagnostic target for oral cancer detection during an oral cavity health assessment [51]. The accurate role of MMP12 protein in the development, invasion, and metastasis of oral carcinoma still needs to be further explored.

### 3.5. MMP12 in Temporomandibular Joint Dysfunction (TMD)

TMD is characterized by restricted jaw movement, joint sound, and pain, frequently involving TMJ disc displacement, internal misalignment, and osteoarthritis (OA). Synovitis is a chronic inflammatory disorder that often coexists with intracapsular abnormalities in TMD [95,96]. Cytokines have been identified as possible mediators of TMD pathogenesis. Previous studies about abnormal human TMJ discs demonstrated a considerable decrease in bilaminar zone elastic fibres [43,97]. Currently, little information is available on the involvement of elastin degradation in the synovitis process in human TMD. MMP12 is an enzyme that can degrade elastin. Furthermore, increasing MMP12 expression activates and may contribute to further increases in elastin-derived peptide levels, resulting in chronic inflammation in the TMJ [43]. The in vivo study conducted by Yoko et al. further verified that MMP12 is related to tissue inflammation and destruction by aggrecanases in TMJ OA mice [44]. Another study conducted by Angelos et al. found that MMP12 expression varies according to the OA severity in various regions of cartilage. In the group with severe OA, osteoclasts expressing MMP12 were also detected [98]. These results indicated that the increased expression of MMP12 in the OA patients’ osteochondral unit implied its potential role in the development of OA.

### 3.6. MMP12 in Other Oral Diseases

Commonly expressed in the oral tissues, MMP12 may be associated with other oral disorders than those mentioned above. MMP12 levels elevated throughout the development of dental periradicular lesions and were associated with periapical tissue loss, including bone resorption [99]. Another study demonstrated that inhibiting MMP12 activity may be critical for non-eruption teeth [100]. MMP12 may also be implicated in the fragmentation and regression of the dental lamina, which seems to play a critical role in preventing the further development of replacement teeth [101]. In addition, MMP12 was proved to be directly engaged in amelogenesis. MMP12 was one of the identified hypomineralization genes that were dysregulated in response to environmental toxicants, resulting in enamel abnormalities [102]. It may be deduced that MMP12 is required for tooth growth and may also be associated with the regulation of inflammatory destruction in oral tissue. Moreover, the oral cavity consists of a diverse and balanced bacterial ecosystem. Once a community has transitioned to a dysbiotic state, numerous oral disorders, such as dental caries, gingivitis, and periodontitis, develop. When challenged with both Gram-negative and Gram-positive bacteria at macrophage-rich entry sites, MMP12 was indicated to show impaired bacterial clearance and aggravated disease progression [2]. In addition, MMP12 could bind to bacterial cell walls, disrupting cellular membranes and ultimately killing the bacterium. However, it remains to be determined whether MMP12 is capable of killing periodontal bacteria. MMP12 is considered a key protein and has critical roles in the physiological and pathological activities of various oral diseases.

## 4. Targeting MMP12 as the Therapeutic Strategy for Oral Diseases

MMP12 and other MMPs are key proteins in keeping oral mucosal integrity and regulating normal biological activities. An imbalance between tissue levels of these proteases and their natural inhibitors is regarded as an important factor in the pathophysiology of various oral diseases, including periodontitis, TMD, and gingival disorders. Therefore, MMP12 might serve as an important molecular target for the diagnosis and treatment of oral diseases. Highly expressed in the tissue of periodontal diseases, TMD, and OSCC, MMP12 may be regarded as the diagnostic marker of these common oral disorders. An excessive MMP12 level in the saliva is related to oral pathological changes. Researchers are allowed to use advanced diagnostic technology to investigate precise chair-side tests or mouth-rinse MMP12 screening tests for monitoring periodontal diseases and other oral disorders [103,104]. Acting as a target to predict periodontal status, cancer, and TMD, reducing the levels of MMP12 in the oral environment may be a possible route to improving periodontal health. In addition, strategies to inhibit the expression of MMP12 and other MMPs have been used in the treatment of periodontal diseases. Interestingly, the effect of MMP12 in the regulation of other MMPs was confirmed using the MMP12 knockout mouse model [64]. Better periodontal status has been correlated with decreased expression of MMP12 in the GCF of patients with juvenile aggressive periodontal disease [54].

The ongoing development of synthetic inhibitors of MMPs may provide opportunities to develop treatment modalities for patients suffering from oral diseases. The synthesis of specific, new MMP12-inhibition targeted drugs to treat oral diseases, featuring fewer side effects than the currently available broad-spectrum MMP inhibitors, is paramount to offer therapeutic benefit without interfering with other MMPs that have an important role in preventing disease progression and resolving tissue destruction [105]. Numerous potent and selective MMP12 inhibitors, such as MMP408, AS111793, and RXP470.1, have been developed and are being tested in various lung diseases in mouse models [106,107,108,109,110]. Another phase I clinical trial is currently registered on Forsee Pharmaceutical’s FP-025, a selective MMP12 inhibitor developed for asthma and COPD [111]. None of these MMP12 inhibitors is yet tested for their efficacy in oral diseases, such as periodontal diseases, TMD, and OSCC. Given the implication of MMP12 in various oral diseases, it will, therefore, be of great interest to see whether disease progression in relevant animal models can be prevented with these specific MMP12 inhibitors. The potential role of MMP12 in oral diseases still needs to be elucidated, and more clinical trials need to find the proper combination of MMP12 inhibitors or their activators.

Repurposing of drugs could be an attractive strategy to search for existing drugs with novel MMP inhibitory activity. For instance, doxycycline, a broad-spectrum antibiotic, has been widely repurposed for the management of various diseases. The sub-lethal concentration of doxycycline was reported to be effective in chronic periodontitis therapy. It was demonstrated to reduce MMP12 activity in vitro [112] and improves the clinical outcome of periodontitis treatment [113]. Nonetheless, antibiotic drug repurposing needs strict monitoring to reduce the risk of antibiotic resistance, despite its safety profile.

In recent years, application of probiotics in oral diseases has attracted much interest. Probiotics are live microorganisms that can maintain microbial balance and confer health benefits on the host by regulating the immune–inflammatory response and restoring the microbial community [114]. Numerous reports have evaluated the effectiveness of oral probiotics as an adjunctive treatment for oral diseases, especially for periodontitis [115,116]. Some reports evidenced that the application of specific strains of probiotics conferred anti-inflammatory and immune-modulating effects in inflammatory disorders related to increased MMP12 levels. *Lactobacillus paracasei* and *L. rhamnosus* were shown to reduce the infiltration of MMP12-expressing inflammatory macrophages into adipose tissue, reducing inflammation and improving host metabolic health [117]. *Bifidobacterium breve* and *L. rhamnosus* were also shown to reduce MMP12 expression in inflamed lung tissue [118]. With that, the application of these probiotic strains could be an effective strategy in targeting the MMP12 expression, inflammatory pathway related to MMP12 synthesis and reducing oral pathogens associated with increased MMP12 expression, hence leading to reduced MMP12 levels and conferring an ameliorative effect on oral diseases. All in all, MMP12 may be considered a potential diagnostic and therapeutic target for various oral diseases.

## 5. Conclusions and Future Directions

MMP12 is one of the critical proteases, with a range of physiological and pathological activities, which has been linked to pro-inflammatory and tissue-remodelling pathways that underpin oral illnesses and tissue remodelling, such as periodontitis, TMD, OSCC, and OTM, as well as bone remodelling. Although animal studies indicate that MMP12 has a critical function in inflammatory and tissue-remodelling pathways in the oral environment, its significance in the pathophysiology of chronic illnesses is not well understood in humans. Additionally, investigations have revealed that MMP12 has a regulatory role in tooth mobility and bone remodelling, albeit the putative regulatory mechanism must be further studied. In the future, selective and effective MMP inhibitors may aid in deciphering the role of specific MMPs, particularly MMP12, in the pathogenesis and therapy of specific oral illnesses. Probiotic interventions targeting MMP12 expression may be viable for improving oral health. The mechanisms of MMP12 in oral disorders are largely unknown. Therefore, more future study is required to elucidate these pathways and aid in developing MMP12-based diagnostic and therapeutic techniques for oral disorders.

## Figures and Tables

**Table 1:** Studies on MMP12 involvement in oral diseases (TMJ, temporomandibular joint; OSCC, oral squamous cell carcinoma; CPD, mild chronic periodontitis; JBO, jaw bone ossification fibroma; DFDBA, demineralized freeze-dried bone allograft; DBBM, deproteinized bovine bone mineral).

Year	Disease Type	Diagnostic Method	Findings	Reference
2017	Closed lock disc disease in the temporomandibular joint	ELISA (Human TMJ synovial fluid)	Increasing MMP12 expression was found in the inflamed TMJ models	[43]
2021	Temporomandibular joint dysfunction	IHC	MMP12 was expressed in the chondrocytes in the superficial zones of the cartilage	[44]
2015	Periodontally compromised teeth	Multiplex bead immunoassay	Significant increase of MMP12, 24 h after activation of periodontitis teeth subjected to orthodontic treatment	[45]
2020	Orthodontic relapse	Quantitative RT-PCR, HE, Masson’s Trichrome staining	Sulforaphane (SFN) reduced the amount of relapse of orthodontic rotation via upregulated MMP12 by increasing gingival elasticity	[46]
2013	Orthodontic tooth movement	GCF multiplexed bead immunoassay	The mean levels of MMP12 were not significantly different between the test and control groups at each time shown	[47]
2017	Orthodontic tooth movement	IHC, Quantitative RT-PCR, micro-CT	Orthodontic tensile strain upregulates MMP12 expression in the tension zone of PDL and induces angiogenesis	[40]
2008	Healthy orthodontic treatment	Quantitative RT-PCR	MMP12 showed more than 100-fold higher expression in PDLs of the second premolars with occlusal contact than the third molars performing a vertical movement	[48]
2021	OSCC, oral submucous fibrosis (OSF)	ELISA (Saliva)	Increased expression of MMP12 appears as the healthy patient advances to OSF and OSCC	[49]
2019	OSCC, CPD, JBO, healthy	ELISA (Saliva), WB, IHF	The levels of MMP12 in the saliva of patients with OSCC were significantly increased compared with those of other groups	[50]
2022	Periodontitis and OSCC	Bioinformatic analysis	MMP12 was identified as one of the hub genes between PD and OSCC	[51]
2017	Periodontitis	Quantitative RT-PCR, IHF, ELISA, WB	The connection of MMP12 production by monocyte-derived cells with CD200/CD200R pathway may play a crucial role in PD progression	[36]
2019	Periodontal disease	ELISA (Saliva)	MMP12 level in saliva reflect different aspects of periodontal inflammation	[52]
2011	Gingival overgrowth periodontitis	Microarray, Quantitative RT-PCR	MMP12 was significantly upregulated in gingival overgrowth tissues of periodontitis patients compared with the healthy control gingival tissues	[53]
2013	Localized aggressive periodontitis	MMP Enzymatic Assay	MMP12 in GCF was reduced significantly up to 6 months, comparable to healthy sites at the same point	[54]
2003	Young healthy patients	Quantitative RT-PCR, WB	No MMP12 expression in odontoblasts or pulp tissue	[42]
2006	Periodontal disease	Quantitative RT-PCR, WB	MMP12 mRNA was induced in the *P. gingivalis*-treated human gingiva fibroblasts	[55]
2021	Mouse calvaria defects	Microarray, Quantitative RT-PCR, IHC	DFDBA and DBBM groups showed a higher mRNA and protein level of MMP12 compared with the control	[56]
2018	Idiopathic gingival fibromatosis	Microarray Quantitative RT-PCR IHC	Low MMP12 expression in the idiopathic gingival fibromatosis	[57]
2016	Aged gingiva	Quantitative RT-PCR, RNA sequencing	MMP12 was upregulated in old gingival tissues, concomitantly with interleukin-1 beta expression	[58]

## Data Availability

The data analyzed during this study are openly available in the GEO database at [https://www.ncbi.nlm.nih.gov/geo/], reference number [GSE161266, GSE164241].
